# Assessing risk from invasive alien plants in China: Reconstructing invasion history and estimating distribution patterns of *Lolium temulentum* and *Aegilops tauschii*

**DOI:** 10.3389/fpls.2023.1113567

**Published:** 2023-02-02

**Authors:** Ming Yang, Haoxiang Zhao, Xiaoqing Xian, Rui Wang, Nianwan Yang, Li Chen, Wan-xue Liu

**Affiliations:** ^1^ State Key Laboratory for Biology of Plant Diseases and Insect Pests, Institute of Plant Protection, Chinese Academy of Agricultural Science, Beijing, China; ^2^ School of Life Sciences, Hebei University, Baoding, China; ^3^ Western Agricultural Research Center, Chinese Academy of Agricultural Sciences, Changji, China

**Keywords:** climate change, distribution patterns, *Lolium temulentum*, *Aegilops tauschii*, overlap area, maximum entropy

## Abstract

**Introduction:**

The establishment of invasive alien plants (IAPs) is primarily driven by climate warming and human activities, and their populations have a negative impact on agricultural economics, ecological systems, and human health. *Lolium temulentum* and *Aegilops tauschii* are critical IAPs in China because they reduce the quality of cereal grains and decrease wheat yields. *Lolium temulentum* is a winter-temperate weed that spreads easily and is poisonous to humans and animals. *Aegilops tauschii* is resistant to herbicides, has a high reproductive rate, and frequently grows in wheat. Both species have been listed in the Ministry of Agriculture and Rural Affairs of the People’s Republic of China’s management catalog since 2006.

**Methods:**

In the present study, the historical occurrence and invasion of each species were collected and reconstructed, which showed that the population outbreak of *L. temulentum* began in 2010, whereas that of *A. tauschii* began in 2000. Using the optimal MaxEnt model, the geographical distributions of *L. temulentum* and *A. tauschii* were predicted based on screened species occurrences and environmental variables under the current and three future scenarios in the 2030s and 2050s (i.e., SSP1-2.6, SSP2-4.5, and SSP5-8.5).

**Results:**

The mean AUC values were 0.867 and 0.931 for *L. temulentum* and *A. tauschii*, respectively. Human influence index (HII), mean temperature of coldest quarter (bio11), and precipitation of coldest quarter (bio19) were the most significant variables for *L. temulentum*, whereas human influence index, temperature seasonality (standard deviation×100) (bio4), and annual mean temperature (bio1) were the critical environmental variables for *A. tauschi.* Suitable habitat areas in China for *L. temulentum* and *A. tauschii* currently covered total areas of 125 × 10^4^ and 235 × 10^4^ km^2^, respectively. Future suitable areas of *L. temulentum* reached the maximum under SSP2-4.5, from 2021 to 2060, whereas for *A. tauschii* they reached the maximum under SSP5-8.5, from 2021 to 2060. Furthermore, the overlap area under the current climate conditions for *L. temulentum* and *A. tauschii* was approximately 90 × 10^4^ km^2^, mainly located in Hubei, Anhui, Jiangsu, Shandong, Henan, Shaanxi, Shanxi, and Hebei. The overlap areas decreased in the 2030s, increased in the 2050s, and reached a maximum under SSP1-2.6 (or SSP2-4.5) with an approximate area of 104 × 10^4^ km^2^. The centroid of *L. temulentum* in Henan was transferred to the southwest, whereas for *A. tauschii* it transferred to higher latitudes in the northeast.

**Discussion:**

Our findings provide a practical reference for the early warning, control, and management of these two destructive IAP populations in China.

## 1 Introduction

Global trade, climate warming, and human-mediated transportation are the primary driving factors for invasive alien plants (IAPs), and these pose a threat to the economy, biodiversity, and human livelihood (Dukes and Mooney, 1999; Pejchar and Mooney, 2009; Diagne et al., 2021). Furthermore, climate warming can alter the geographical distribution of IAPs and increase their establishment rates, thereby facilitating the global spread of IAPs (Petitpierre et al., 2012; Bellard et al., 2013). The Intergovernmental Panel on Climate Change (IPCC) issued the sixth assessment report (AR6), indicating that temperature and global carbon dioxide (CO_2_) emissions will gradually increase under five Shared Socioeconomic Pathways (SSPs), namely SSP1-1.9, SSP1-2.6, SSP2-4.5, SSP3-7.0, and SSP5-8.5. Under the most optimistic scenarios, the global temperature would rise by 1.5 °C from 2050 to 2100, whereas under the worst-case emission scenario, the temperature would rise by 4.4 °C during this period; in either case, the warming would expand the geographical distribution of IAPs (Bellard et al., 2013; IPCC, 2021). Human activities, such as land use, urbanization, and trade transport, promote the invasion of IAPs through seed dispersal and disruption to native plant habitats (Vilà and Ibáñez, 2011; Rew et al., 2018). Furthermore, when improving trade contacts and construction economics, humans inevitably try to establish better dispersal corridors to transport plants or others items; however, these new pathways may detrimentally facilitate the spread of non-native plants to a new region as some seeds may be attached to the items being transported (Kueffer, 2017). Population growth and agricultural development are drivers of land use expansion and urbanization, which disturb native ecosystems, and inherently complement the wide niche of invasive plant establishment and colonization (Le Maitre et al., 2014). Therefore, estimating the distribution patterns of IAPs that are driven primarily by climate warming and human activities could improve the understanding of the spread of IAPs and help in the implementation of advanced control and management strategies worldwide.

As the third most plant-diverse country in the world, China has reported 402 IAPs, which threaten China’s rich biodiversity, disrupt ecosystem function, and even cause huge economic losses (Yan et al., 2012; Ma, 2020). Most IAPs with high reproduction and adaptation rates compete for light and nutrients and crowd out native species, leading to decreased biodiversity and genetic diversity (Keller and Shea, 2021). Previous studies have shown that invasive plant populations that outbreak in new regions of China are difficult to control, decrease the value of native species, and create economic losses in the order of 4.52–67.71 billion on field and wetland ecosystems and more than US$14.5 billion on pulling and pesticide development per year (Xu et al., 2006; Yan et al., 2012). Poaceae comprise the third most populated IAP in China, with half of the Poaceae family transported through foraging, and the remainder through cereal trade, including *Lolium temulentum* and *Aegilops tauschii* (Zhang et al., 2021a).

*Lolium temulentum*, known as darnel, is an annual C_3_ winter weed that is native to the Mediterranean and is widely distributed throughout North America, Asia, Europe, South America, Africa, and Oceania, in over 60 countries and 120 regions (Lush, 1976; Sarno et al., 1986; CABI, 2022). It was first recorded in China in 1954 in a shipment of wheat imported from Bulgaria, and has since spread to Southwest and Eastern China. *Lolium temulentum* has strong environmental adaptability under low temperature and high nitrogen (N), kalium (K), phosphorus (P) conditions, a high reproductive rate with a fibrous root system, and 6–30 terminal spikes that each spike with 7–9 nerved, mainly reproducing *via* seed (Terreix, 1968; Thomas et al., 2011). Darnel typically grows amongst crops of wheat and other winter cereals and has a similar size and weight to wheat, which makes it difficult to separate. Therefore, its presence leads to a decline in crop quality and causes yield loss (Ratera, 1983). The mix rate of darnel to wheat has been positively correlated with a reduction in wheat yield (Lin et al., 2004). In addition, darnel has three poisonous compounds, namely temulin and loline in the seeds and perloline in the stem (Swallen, 1961; Dannhardt and Steindl, 1985). When consumed with mixed wheat, these may cause dizziness, vomiting, and even death in humans and livestock (Liu et al., 2010). Previous studies have reported that, in addition to wheat losses, human poisoning has occurred in every province of China, including Shaanxi, Jiangsu, Heilongjiang, and Hubei, since the invasion of *L. temulentum* in the 1900s (Lin et al., 2004; Zhou et al., 2007).

*Aegilops tauschii*, is a noxious weed native to Eastern Europe and Western Asia that has subsequently spread in France, Italy, Turkey, the United States, and Mexico (Donald and Ogg, 1991; Fang et al., 2015). In 1955, it was found in China’s Henan Province, while now it is located in Shandong, Shanxi, Hebei, Henan, and Anhui. *Aegilops tauschii* typically grows with wheat, has a high reproductive rate with 5–13 terminal spikes, where each spike has 7–9 nerved *via* seed reproduction. Wheat and *A. tauschii* have similar seed sizes and germination and emergence conditions, which make it difficult to completely eradicate. The presence of *A. tauschii* causes wheat yield loss and a decrease in quality (Gong, 2022). This species has pesticide resistance, and the lack of new pesticides to control or destroy *A. tauschii* has facilitated the reckless spread of this IAP among wheat crops (Wang et al., 2021). One study showed a wheat yield decrease of 15–20%, whereas the worst situation attained a loss of 50% when *A. tauschii* was mixed with wheat crops, which mainly affected Henan, Shandong, and Shaanxi (Gao et al., 2021a; Wang et al., 2021).

*Lolium temulentum* and *A. tauschii* have continued to spread to new environments in provinces throughout China, and poses a potential threat to crops, primarily cereals (Wu et al., 2022). Both *L. temulentum* and *A. tauschii* are considered the most prolific weeds in wheat production industry and are listed in the “List of imported plant quarantine pests of the People’s Republic of China”, “National List of Invasive Alien Species under Key Management (First Batch)”, and “List of administrative regions where agricultural phytosanitary pests are distributed nationwide” by the Ministry of Agriculture and Rural Affairs in 2007, 2013, and 2016, respectively.

Species distribution models (SDMs), such as maximum entropy (MaxEnt), the genetic algorithm for rule-set prediction (GARP), and biomood_2_, have become important tools to predict spatial species distributions through mathematical algorithms consisting of species occurrence (or presence-absence) data and environmental variables (Peterson et al., 2011; Sillero, 2011). MaxEnt uses presence data alone, and provides efficient algorithms with optimal performance, toilless analysis, and avoids overfitting on other SDMs (Elith et al., 2006; Phillips et al., 2006; Phillips and Dudík, 2008). Previous studies on IAPs, including *Prosopis juliflora*, *Ageratina adenophora*, and *Cenchrus spinifex*, used the MaxEnt model to predict geographical distribution patterns (Cao et al., 2021; Singh et al., 2021; Xian et al., 2022). However, the default MaxEnt model has some methodological issues in balancing goodness-of-fit with model complexity, which leads to overfitting and poor transferability when projected in a novel environment (Warren and Seifert, 2011; Guevara et al., 2018). There are some ways to optimal MaxEnt parameters, such as ENMeval, SDMtune, and kuenm. Compared with other ways, ENMeval is widely used to avoid model overfitting *via* automatic matching of model parameter combinations (Muscarella et al., 2014). Therefore, based on Rstudio in the R software, ENMeval was developed to address the noise in the data and reduce biased sampling or evaluation methods in the default algorithm *via* tuning the settings for specific species and calibrating the data sets (Merow et al., 2014; Muscarella et al., 2014; Kass et al., 2021).

*Lolium temulentum* and *A. tauschii* are critical quarantine weeds in China, causing wheat yield reduction. However, the suitable and overlapping areas of two species are still unkown. Therefore, predicting the potential geographical distribution of *L. temulentum* and *A. tauschii* to provide a theoretical basis for future surveillance and control. In this study, we used the optimal MaxEnt to predict the geographical distribution of *L. temulentum* and *A. tauschii* throughout China under current and future climate scenarios. The steps included (1) reconstructing the historical invasion of *L. temulentum* and *A. tauschii* using ArcGIS software; and (2) predicting potential geographical distribution under the current, 2030s, and 2050s climate scenarios using the optimal MaxEnt model; (3) calculating future special changes and centroid transfer; (4) analyzing the overlap between *L. temulentum* and *A. tauschii* under the current and future climate scenarios; and (5) identifying significant environmental variables. Our results provide a foundation for the development of early warning, prevention, control measures, and co-management for *L. temulentum* and *A. tauschii* in China under predicted future climate change scenarios.

## 2 Material and methods

### 2.1 Occurrence records

**Figure 1:**
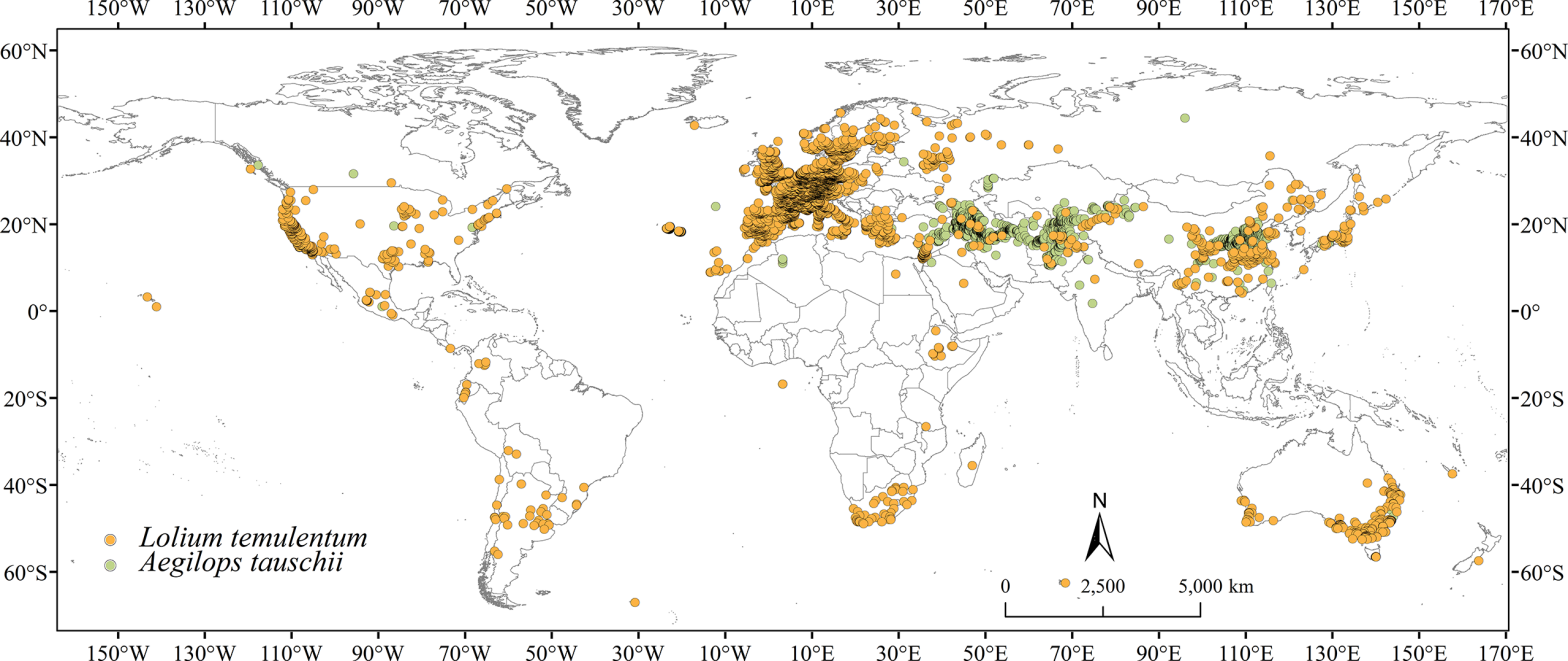
Occurrence records of *Lolium temulentum* and *Aegilops tauschii* worldwide.

*Lolium temulentum* occurrence records were collected from the Global Biodiversity Information Facility (GBIF.org, 2022a), the Chinese Virtual Herbarium (CVH; https://www.cvh.ac.cn/, accessed May 2022), the Ministry of Agriculture and Rural Affairs of the People′s Republic of China (http://www.moa.gov.cn/, accessed May 2022), and China National Knowledge Infrastructure (CNKI; https://www.cnki.net/). *Aegilops tauschii* occurrence records were downloaded from GBIF (GBIF.org, 2022b), CVH in August 2022, and CNKI. For non-longitude and latitude records, Google Earth was used to obtain precise coordinates. To decrease the effect of duplicate coordinates and sampling bias, all occurrence records for both species were filtered using ENMTools to ensure that only one record was retained in per 5 km × 5 km grid square (**Figure 1**). Ultimately, 4578 and 1966 occurrence records of *L. temulentum* and *A. tauschii* were retained worldwide, respectively.

### 2.2 Invasive historical reconstruction

*Lolium temulentum* and *A. tauschii* occurrence records were collected from CNKI and CVH. The year in which the earliest published articles or specimens were published was regarded as the invasive time in China. There were 217 for *L. temulentum* and 694 points for *A. tauschii* in China from 1950 to 2022. We used ArcGIS 10.5 was used with the GCS_Beijing_1954 geographic coordinate system, to map the invasive historical process of *L. temulentum* and *A. tauschii* over a 20-years interval in China.

### 2.3 Environmental variables

Current climate data that comprised 19 bioclimatic variables at 2.5 arc-min resolution from 1979 to 2013 were downloaded from PaleoClim v1.2 (http://www.paleoclim.org/) (Brown et al., 2018). Future climate and elevation data with 2.5 arc-min resolution were obtained from WorldClim v. 2.0 (https://www.worldclim.org/), and these included two time periods (2021–2040 and 2041–2060) and three climate scenarios (SSP1-2.6, SSP2-4.5, and SSP5-8.5) based on the BCC-CSM2-MR model (Eyring et al., 2016; Fick and Hijmans, 2017). Slope and Aspect were extracted from elevation data using ArcGIS software. Soil data (AWC_CLASS, T_OC, T_PH_H_2_O, and T_SAND) were downloaded from the Harmonized World Soil Database version 1.2 (https://www.fao.org/) (Fischer et al., 2008). The Global Human Influence Index (HII) from 1995 to 2004 was downloaded from the Socioeconomic Data and Applications Center (https://sedac.ciesin.columbia.edu/) (Wildlife Conservation Society and Center for International Earth Science Information Network, 2005). A total of 27 environmental variables are listed in **Table S1**.

The collinearity of environmental variables can influence parameter optimization during MaxEnt model calibration (Dormann et al., 2013). To address this issue, the “band collection statistics” in ArcGIS was used to estimate the linear correlations and eliminate the highly linear correlations of 27 environmental variables (**Figure S1**). All species occurrences of *L. temulentum* and *A. tauschii* and related environmental variables were entered into MaxEnt 3.4.4 to establish initial SDMs that provide percent contributions of each environmental variable. Comparing the correlation between two environmental variables (|r| > 0.8), the lower percent contribution was removed. Finally, 11 and 12 environmental variables screened for *L. temulentum* and *A. tauschii* are shown in **Figure S2**.

### 2.4 Model calibration, settings, and evaluation

SDMs, such as the MaxEnt model, face the challenge of balancing goodness-of-fit with model complexity because of their over-reliance on default settings (Warren and Seifert, 2011). Therefore, we used the R package (ENMeval) to calibrate the MaxEnt model to address these issues (Muscarella et al., 2014; Kass et al., 2021). Feature combinations (FCs) and regularization multipliers (RMs) are regarded as critical parameters for optimal settings in model calibration (Muscarella et al., 2014). RMs act synergistically in all FCs, which can adjust the level of regularization through each RM to protect against overfitting (Phillips, 2008). Based on the occurrence records and environmental variables of *L. temulentum* and *A. tauschii*, RM was set from 0.5 to 4, with a 0.5 interval, and six different FC combinations, including L, LQ, LQP, LQH, LQHP, and LQHPT (L = linear, H = hinge, Q = quadratic, P = product, and T= threshold) (Phillips and Dudík, 2008). The Akaike Information Criterion corrected (AICc) reflects the goodness-of-fit and model complexity, and the minimum AICc (i.e., Δ AICc = 0) was regarded as the best model parameter (Warren and Seifert, 2011). Therefore, for 48 combinations of FCs and RM values, FCs were set as LQHPT and RM was set to 0.5.

Optimized occurrence records and environmental variables of *L. temulentum* and *A. tauschii* were entered into MaxEnt 3.3.4; 25% of occurrence records were selected as training data, whereas the other 75% were considered testing data. The optimal MaxEnt model was set to replicate 10 times, and the replicated run type was set to “Bootstrap”. The maximum number of background points was set to 10000, and the output format was set to “Cloglog”, “Random seed” was chosen to improve the model’s randomness, and other parameters were left as default settings.

The area under the curve (AUC) of the receiver operator characteristic (ROC) is a metric for model performance (Roca et al., 2004). The AUC value is closely related to the rates of negative and positive values. The values range from 0 to 1, and those that are lower than 0.6 indicate that the model is unreliable. AUC values have been classified into four indices: poor (0.6 ≤ P < 0.7), moderate (0.7 ≤ P < 0.8), good (0.8 ≤ P < 0.9), and excellent (0.9 ≤ P < 1) (Swets, 1988).

### 2.5 Centroid transfer and threshold chosen

The centroid of change trend in geographical distribution from the current climate to future scenarios was calculated using the ArcGIS software. In this study, we focused on the centroid transfer of *L. temulentum* and *A. tauschii* in the 2030s and 2050s, for example, SSP1-2.6, SSP2-4.5, and SSP5-8.5. The geographical distributions of *L. temulentum* and *A. tauschii* were reclassified and transferred to a vector file that dissolved a single central point to describe the direction (longitude and latitude) of the predicted change from the current climate to future scenarios in the 2030s and 2050s. This allows the model to depict shifts in habitat by tracking the centroid transfer and transfer distance.

The potential geographical distribution of *L. temulentum* and *A. tauschii* in a particular raster was based on MaxEnt model output. The maximum test sensitivity plus specificity was chosen as the threshold (Cantor et al., 1999; Manel et al., 2001). The potential geographical distribution of *L. temulentum* was classified into the following four categories under current and future climates: unsuitable (P < 0.35), poor (0.35 ≤ P < 0.5), moderate (0.5 ≤ P < 0.7), and high (0.7 ≤ P < 1). Similarly, *A. tauschii* was also classified into the following categories: unsuitable (P < 0.25), poor (0.25 ≤ P < 0.4), moderate (0.4 ≤ P < 0.6), and high (0.6 ≤ P < 1). Furthermore, ArcGIS was used to calculate the niche overlap of *L. temulentum* and *A. tauschii* under the current climate and three future scenarios in the 2030s and 2050s. The threshold of binary classification was based on the maximum test sensitivity plus specificity, for *L. temulentum*: unsuitable (P < 0.35) and suitable (0.35 ≤ P < 1), and for *A. tauschii*: unsuitable (P < 0.25) and suitable (0.25 ≤ P < 1).

### 2.6 Niche breadth and niche overlap

Niche breadth is the range and variety of living conditions in a species’ niche, such as temperature range and food variety (Sexton et al., 2017). The value of niche breadth was calculated using ENMTools, Levins’ B1 and B2, that is, inverse concentration and uncertainty, respectively (Levins, 1968; Warren et al., 2010). The values of Levins’ B1 and B2 ranged from 0 to 1, indicating narrow to wide niche breadth (Levins, 1968). Furthermore, based on the ENMTools, there was value in evaluating the range of niche overlap in different species (Schoener’s *D*) (Schoener, 1968; Warren et al., 2008). The value of Schoener’s *D* ranged from 0 to 1, indicating that no niche overlapped to niche overlap identically (Warren et al., 2008). This could be directly compared to the traditional ecological assessment of niche overlap, including the overlap of microhabitat and diet.

## 3 Results

### 3.1 Model performance

For *L. temulentum* and *A. tauschii*, Δ AICc was calculated 400.24 and 254.95 in default MaxEnt model (FC = LQHPT and RM = 1). The optimal MaxEnt parameter settings for predicting the potential geographical distribution of *L. temulentum* and *A. tauschii* were when RM was set to 0.5, and FC was set as LQHPT *via* model calibration, Δ AICc was calculated 0. The mean AUC of *L. temulentum* and *A. tauschii* was 0.867 and 0.931, indicating the MaxEnt performance was “good” and “excellent”, respectively (**Figure S3**).

### 3.2 Invasive historical reconstruction

**Figure 2:**
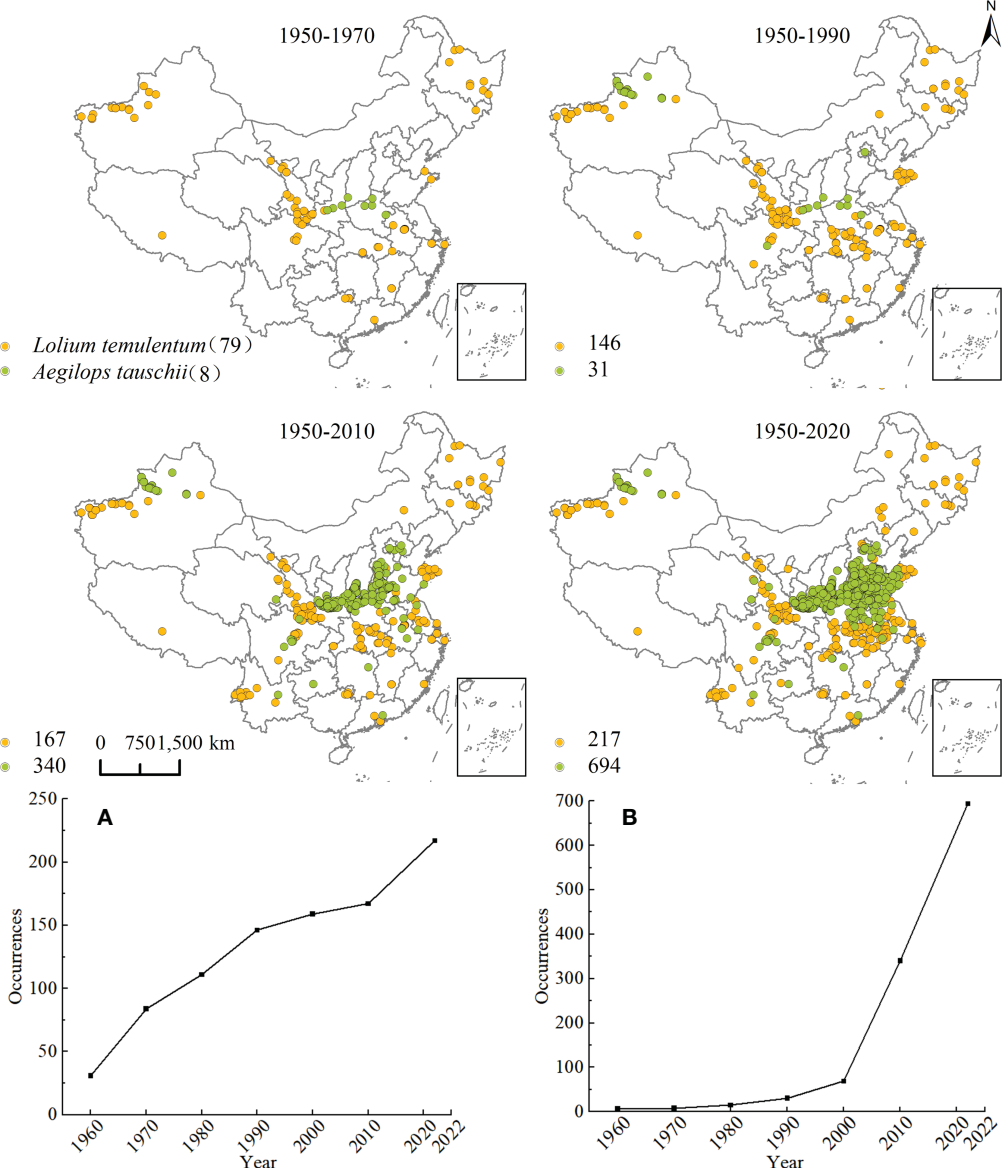
Historical reconstruction of the expansion process and increased species occurrence of **(A)** *Lolium temulentum* and **(B)** *Aegilops tauschii* from 1950 to 2022.

The reconstruction of the historical expansion of *L. temulentum* and *A. tauschii* is shown in **Figure 2**. The impact on the cereal trade from the spread of *L. temulentum* into China’s Heilongjiang province was first documented in 1956. Subsequently, *L. temulentum* gradually spread to most provinces, primarily in northwestern Xinjiang, the border of Gansu, Shaanxi, and Sichuan, and with a scattered distribution in Jilin, Shandong, Zhejiang, Anhui, Shanghai, Hubei, Jiangxi, Guangxi, Guangdong, and Tibet. Until 1990, the spread of *L. temulentum* increased in Qinghai, Jiangsu, Henan, Fujian, and Inner Mongolia, with the most notable increases in Hubei and Shaanxi. In the 2010s, the spread of *L. temulentum* increased in Hebei and Yunnan, with more increases in Yunnan and Jiangsu. To date, Liaoning, Tianjin, Beijing, Chongqing, Guizhou, and Ningxia are the most invaded regions of *L. temulentum*, which is primarily located in Gansu, Xinjiang, Yunnan, Hubei, Henan, Anhui, and Shandong. The historical spreading trend of *L. temulentum* began in Northwest China, spread to the Central China surrounding that region, then spread to North China, and sporadically throughout the Southwest and South China.

In 1950, it was reported that *A. tauschii* spread in Henan as the source of the D-gene in wheat, and spread sporadically in Shanxi and Shaanxi. Until 1990, *A. tauschii* had spread gradually in Xinjiang, Sichuan, and Beijing; however, by the 2010s, *A. tauschii* had spread to Jiangsu, Shaanxi, Guizhou, Hubei, Henan, Yunnan, Shandong, Shanxi, Guangdong, Anhui, and Gansu, primarily located on the border of Hubei, Henan, Shandong, and Shaanxi. Until now, Qinghai and Tianjin had become increasingly invaded regions, primarily in the Shaanxi, Shanxi, Hubei, Henan, Shandong, and Anhui regions. The historical trend shows the spread of *A. tauschii* originating in the southeast and northwest areas of Northwest China and spreading to the border of North China and down to Central and Eastern China. The spread continued constantly to the surrounding provinces but occurred more sporadically in areas in Southwest China.

The trends associated with species occurrence for *L. temulentum* and *A. tauschii* for the period from 1950 to 2022 are shown in **Figure 2**. The number of *L. temulentum* increased rapidly from 1950 to 1990, but then declined until 2010 because of global control strategies. In 2010, the rate of increase for *L. temulentum* recovered, and ultimately exceeded the rate from the initial period. The number of *A. tauschii* increased slowly after the introduction of the species; however, in the 2000s, its occurrence increased significantly.

### 3.3 Potential geographical distribution under the current and future climate scenarios

**Figure 3:**
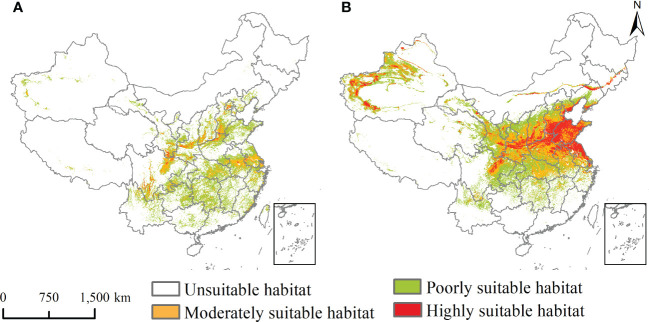
Potential geographical distribution of **(A)** *Lolium temulentum* and **(B)** *Aegilops tauschii* under the current climate.

**Figure 4:**
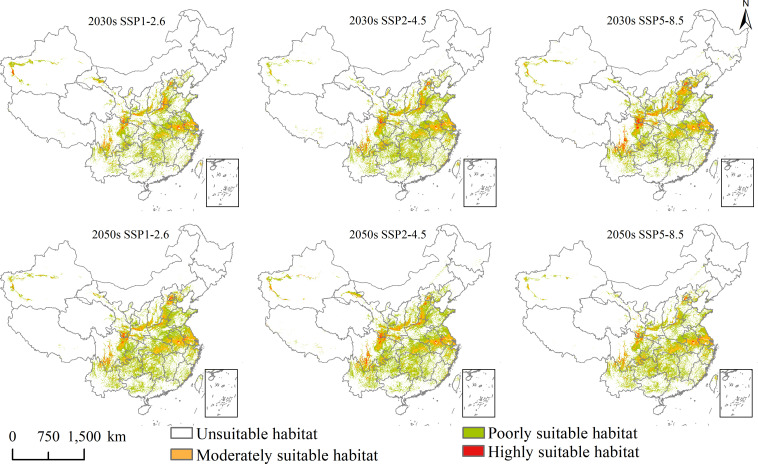
Future potential geographical distribution of *Lolium temulentum* under three scenarios in the 2030s and 2050s.

**Figure 5:**
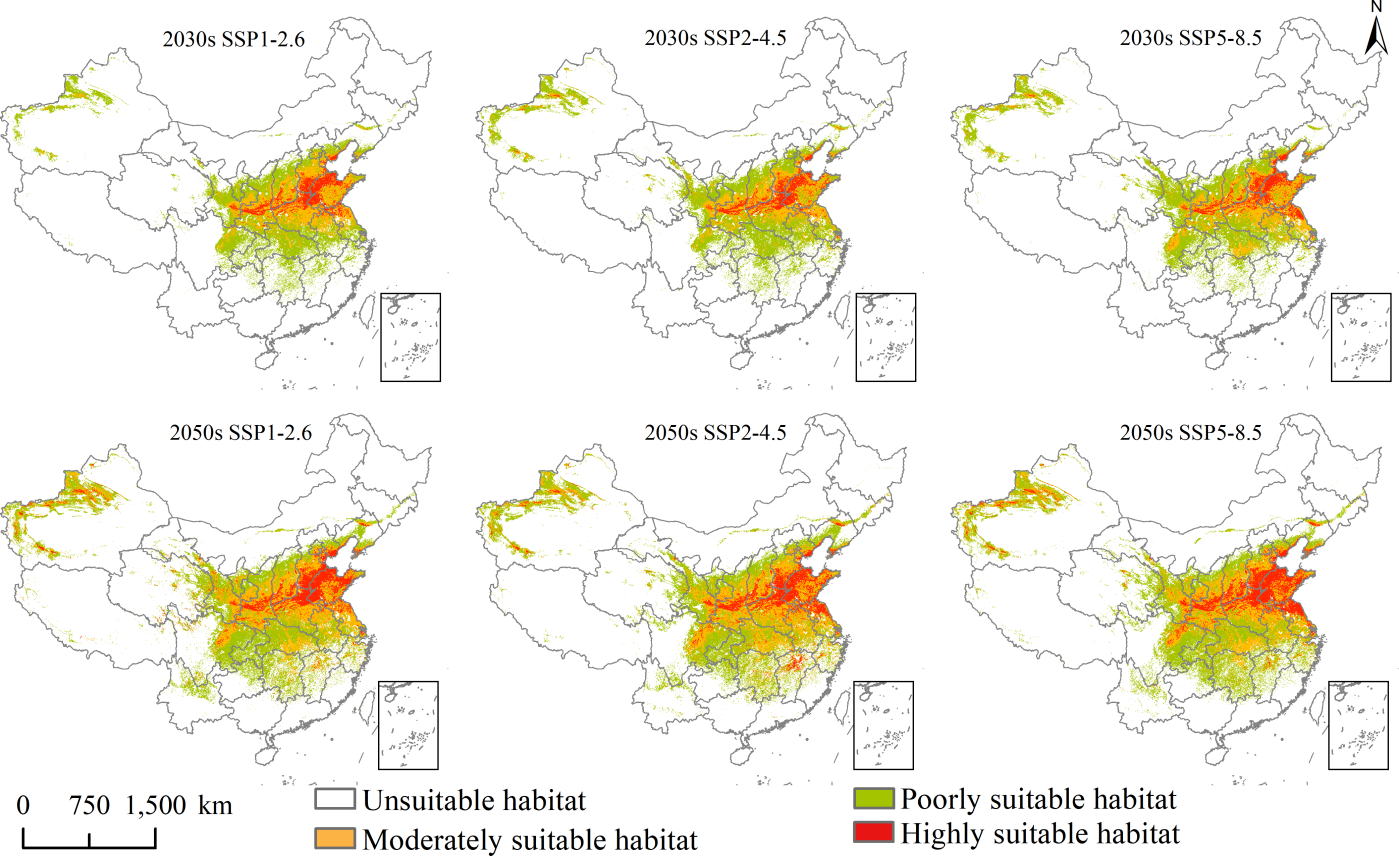
Future potential geographical distribution of *Aegilops tauschii* under three scenarios in the 2030s and 2050s.

The potential geographical distribution of *L. temulentum* and *A. tauschii* for the current climate conditions in China is shown in **Figure 3**, and under the 2030s and 2050s three climate scenarios, namely SSP1-2.6, SSP2-4.5, and SSP5-8.5, are presented in **Figures 4** and **5**. The mainly suitable habitats under the current and future climate scenarios for *L. temulentum* were mainly located in northern Shanghai, southwestern and northeastern Sichuan, central Anhui, western Jiangsu, southern Gansu, central and southern Shaanxi, southwestern Shanxi, northern Henan, southern Hebei, and southern Beijing, Hubei, Tianjin, and Xinjiang.

The mainly suitable habitats under the current and future climate scenarios for *A. tauschii* were located primarily in northeastern Sichuan, central and southeastern Gansu, central Ningxia, central Shaanxi, northern and eastern Henan, northern Anhui, northern Shanghai, northern Jiangsu, Shandong, southern Hebei, southwestern Shanxi, southern Tianjin, central Beijing, northern and southern Liaoning, central Jilin, central Inner Mongolia, northwestern and southwestern Xinjiang, and Hubei.

In the current climate, the current total, highly, and moderately suitable habitat areas of *L. temulentum* were up to 125 × 10^4^, 2 × 10^4^, and 29 × 10^4^ km^2^, accounting for 13.02, 0.21, and 3.02% of the total area of China, respectively. For *A. tauschii*, the current total, highly, and moderately suitable habitat areas were up to 235 × 10^4^, 47 × 10^4^, and 72 × 10^4^ km^2^, accounting for 24.48, 4.90, and 7.50% of the total area of China, respectively.

Under SSP1-2.6, SSP2-4.5, and SSP5-8.5 in the 2030s, for *L. temulentum*, the overall trend in total, highly, and moderately suitable habitat areas of *L. temulentum* increased. The total and highly suitable habitat areas of *L. temulentum* reached the maximum under SSP2-4.5 and SSP5-8.5, up to 133 × 10^4^ and 5 × 10^4^ km^2^, accounting for 13.85 and 0.52% of the total area of China, respectively. For *A. tauschii*, the overall trend in total and highly suitable habitat areas of *A. tauschii* decreased. The total and highly suitable habitat areas reached the maximum under SSP5-8.5, up to 168 × 10^4^ and 19 × 10^4^ km^2^, accounting for 13.85 and 0.52% of the total area of China, respectively.

Under SSP1-2.6, SSP2-4.5, and SSP5-8.5 in the 2050s, for *L. temulentum*, the overall trend in total, highly, and moderately suitable habitat areas of *L. temulentum* increased. The total and highly suitable habitat areas of *L. temulentum* reached the maximum under SSP2-4.5, up to 144 × 10^4^ and 4 × 10^4^ km^2^, accounting for 15.00 and 0.42% of the total area of China, respectively. For *A. tauschii*, the overall trend in total and highly suitable habitat areas of *A. tauschii* increased. The total and highly suitable habitat areas reached the maximum under SSP5-8.5 and SSP2-4.5, up to 239 × 10^4^ and 40 × 10^4^ km^2^, accounting for 24.89 and 4.17% of the total area of China, respectively.

### 3.4 Future spatial changes and centroid transfer

**Figure 6:**
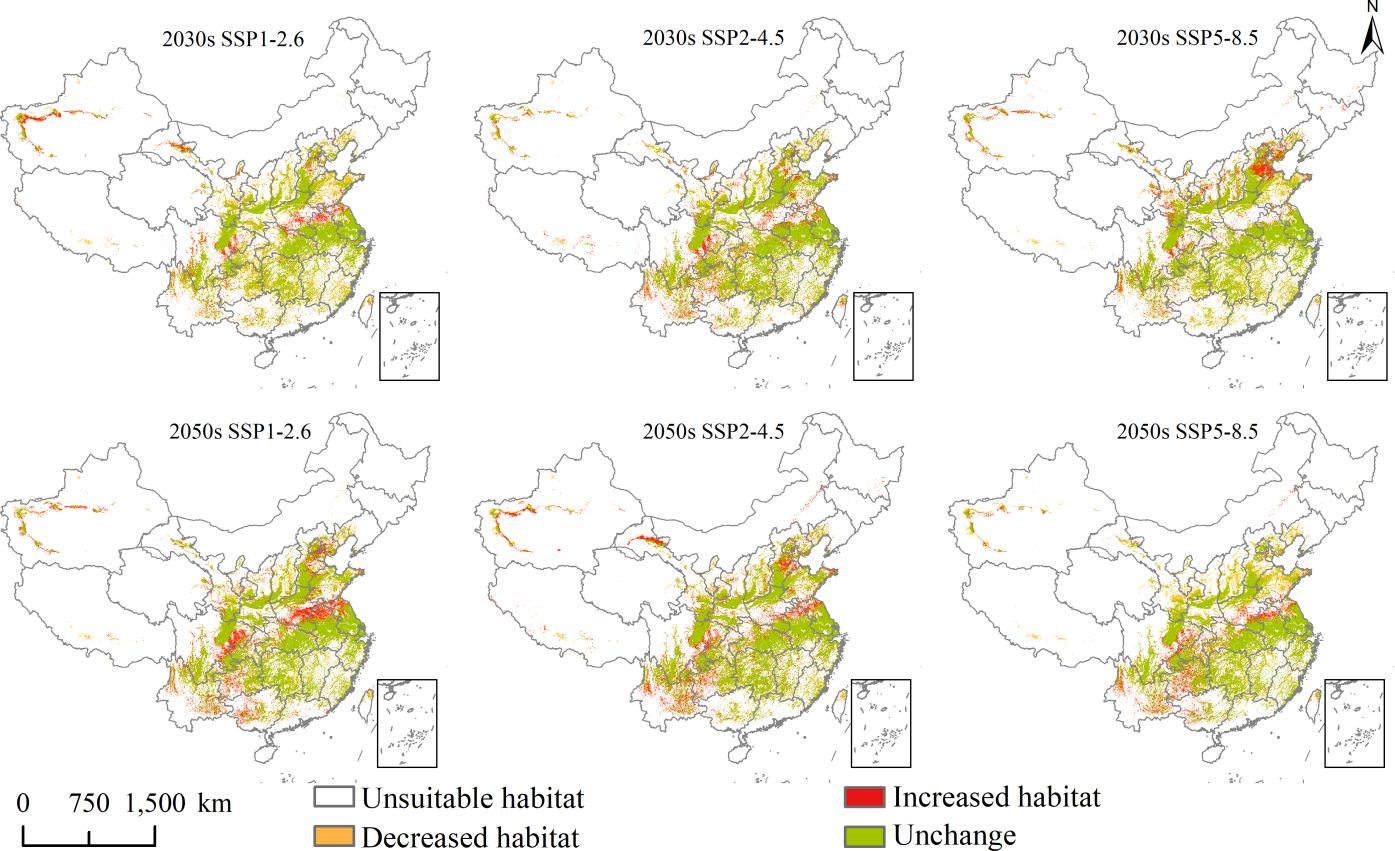
Future potential geographical distribution change of *Lolium temulentum* under three scenarios in the 2030s and 2050s.

**Figure 7:**
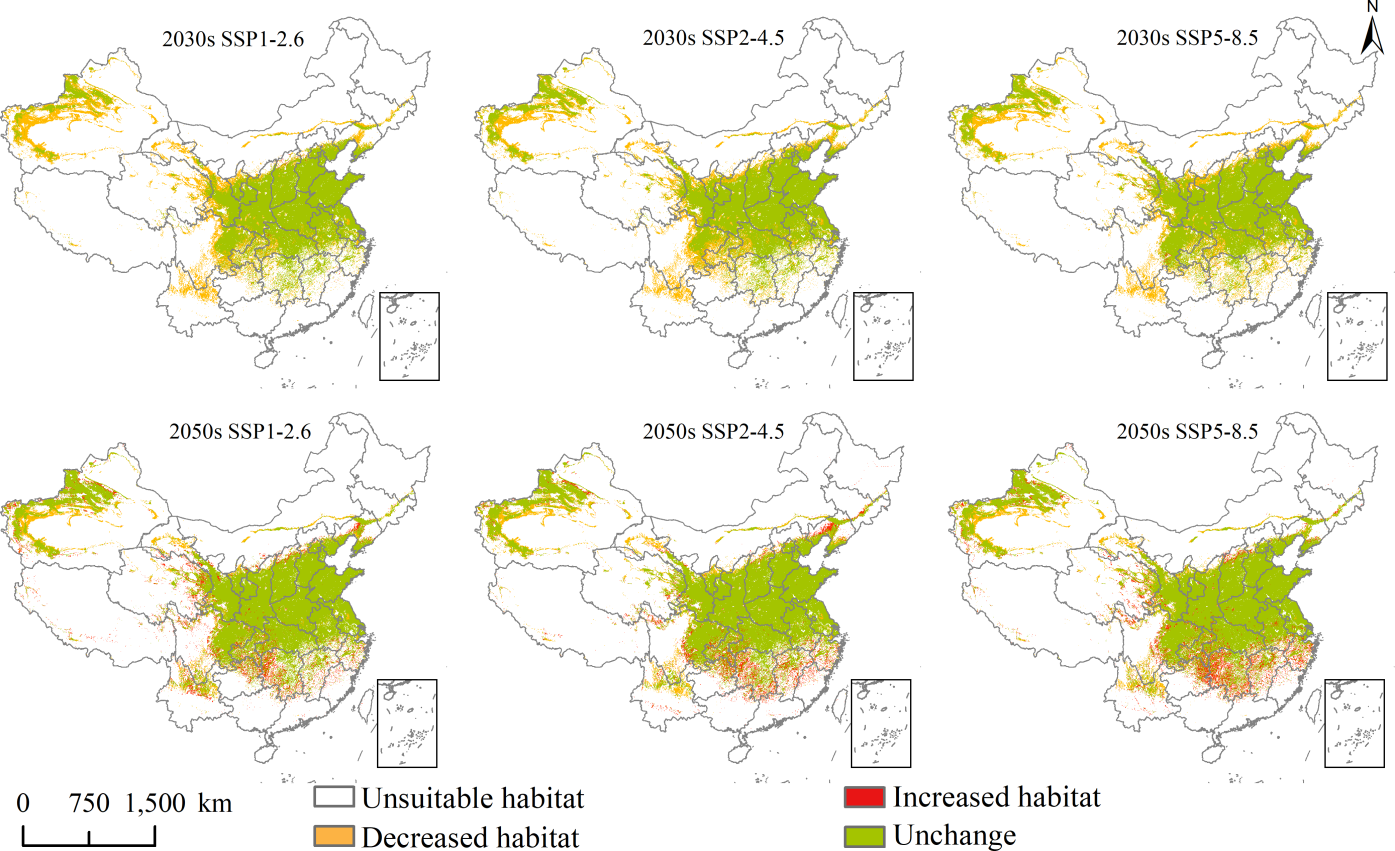
Future potential geographical distribution change of *Aegilops tauschii* under three scenarios in the 2030s and 2050s.

Changes in the future potential geographical distribution of *L. temulentum* and *A. tauschii* under SSP1-2.6, SSP2-4.5, and SSP5-8.5 in the 2030s and 2050s were shown in **Figures 6** and **7**. Under the three scenarios in the 2030s, the increased habitat areas of *L. temulentum* in China were up to 32 × 10^4^, 17 × 10^4^, and 21 × 10^4^ km^2^, mainly located in eastern Sichuan, northern and western Shandong, eastern Hebei, and northwestern Xinjiang, sporadically distributed in Southwest, Central, and Easter of China, and decreased habitat areas were up to 32 × 10^4^, 17 × 10^4^, and 21 × 10^4^ km^2^, mainly located in northeastern and southeastern Guizhou, southern Shaanxi, southeastern Gansu, central Shanxi, and northern Shandong, sporadically distributed in Southwest and Central China. In the 2050s, the increased habitat areas of *L. temulentum* in China were up to 23 × 10^4^, 22 × 10^4^, and 28 × 10^4^ km^2^, mainly distributed in northwestern and southeastern Yunnan, central Guizhou, eastern Sichuan, northern Anhui, southern Henan, central Jiangsu, and western Xinjiang, with decreased habitat areas of 23 × 10^4^, 26 × 10^4^, and 23 × 10^4^ km^2^, sporadically distributed in eastern and western Southwest, Central, and southern North China.

Under the three scenarios in the 2030s, the increased habitat areas of *A. tauschii* in China were up to 1 × 10^4^, 0 × 10^4^, and 1 × 10^4^ km^2^, sporadically located in eastern Southwest and southern North China, and decreased habitat areas were up to 74 × 10^4^, 73 × 10^4^, and 68 × 10^4^ km^2^, mainly located in northeastern Yunnan, southern and eastern Sichuan, southeastern Qinghai, southern and central Gansu, northwestern Ningxia, southwestern Liaoning, eastern Jilin, central Inner Mongolia, and northwestern and southwestern Xinjiang. In the 2050s, the increased habitat areas of *A. tauschii* in China were up to 23 × 10^4^, 22 × 10^4^, and 28 × 10^4^ km^2^, and sporadically located in Yunnan, Sichuan, Chongqing, Guizhou, Hunan, Hunan, Jiangxi, Zhejiang, Anhui, Hubei, Qinghai, Shanxi, and Xinjiang, and the decreased habitat areas were up to 23 × 10^4^, 26 × 10^4^, and 23 × 10^4^ km^2^, mainly located in northeastern Yunnan, southern Sichuan, northern Gansu, northern Ningxia, and northwestern and southwestern Xinjiang, and sporadically distributed in Tibet, Qinghai, Inner Mongolia and Liaoning.

The trend of centroid transfer of *L. temulentum* and *A. tauschii* in China from the current climate to the three scenarios (SSP 2.6, SSP 4.5, and SSP 8.5) in the 2030s and 2050s is shown in **Figure S4**. For *L. temulentum*, the centroid transferred to northeastern and northwestern in China from current to 2030s in the three scenarios, and moved to southeastern and southwestern under the 2050s in the three scenarios (**Figure 3B**). The longest transfer distance of *L. temulentum* centroid from the current climate to all future scenarios in the 2030s and 2050s was approximately 766.54 km and occurred under SSP5-8.5 in the 2030s. For *A. tauschii*, the centroid transferred to northeastern in China from current to 2030s in the three scenarios, and moved to southeastern and southern under the 2050s in the three scenarios (**Figure 3A**). The longest transfer distance of the centroid of *A. tauschii* from the current climate to the all future scenarios in the 2030s and 2050s was approximately 780.87 km under SSP1-2.6 in the 2050s.

### 3.5 Niche breadth and niche overlap

Using ENMTools, the niche breadths of *L. temulentum* and *A. tauschii* were determined. The B2 niche breadth value of *L. temulentum* was 0.9308, which was similar to the value of *A. tauschii* (B2 = 0.9006). These values indicate that *L. temulentum* and *A. tauschii* are likely to fit a broad niche. However, the *D* value was 0.4742, suggesting only moderate niche overlap between *L. temulentum* and *A. tauschii*.

**Figure 8:**
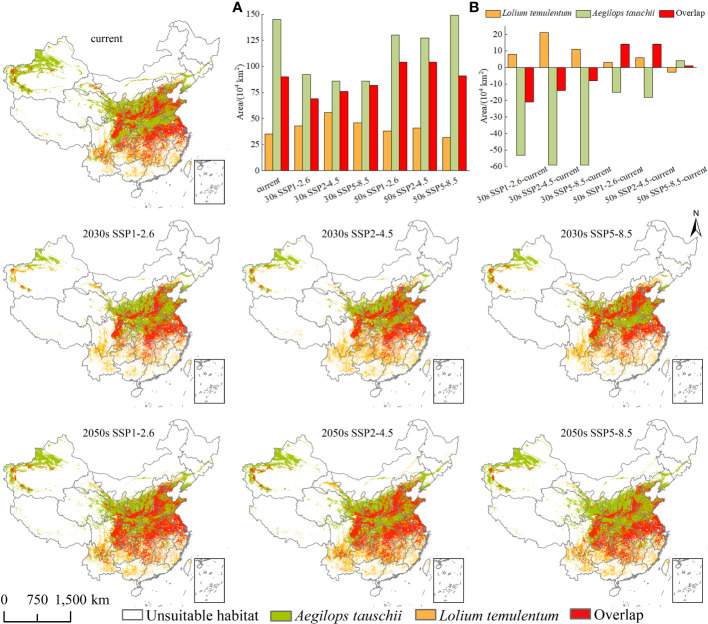
The niche overlap of *Lolium temulentum* and *Aegilops tauschii* under the current climate and future scenarios in the 2030s and 2050s, **(A)** the single plant and overlap areas, **(B)** the change of single plant and overlap areas from the current climate to the future scenarios.

Under the current climate, there are four scenarios, namely unsuitable habitat, the geographical distribution of either *L. temulentum* or *A. tauschii*, and the overlap of the two (**Figure 8**). The overlap area was 90 × 10^4^ km^2^, and was primarily located in northern Yunnan, southern and northeastern Sichuan, northern Guizhou, southern Chongqing, Hunan, Jiangxi, northern Zhejiang, Shanghai, southeastern Hubei, central Anhui, southern Jiangsu, northern Shandong, northern and southern Henan, central and southwestern Shaanxi, southern and northeastern Gansu, southwestern and central Shanxi, southern Hebei, Beijing, southwestern Liaoning, and southern Xinjiang. The remaining areas of *L. temulentum* and *A. tauschii* were 35 × 10^4^ and 145 × 10^4^ km^2^, respectively.

The future overlap between *L. temulentum* and *A. tauschii* is shown in **Figure 8**. Compared with the current overlap area, the overlap area of both under the three scenarios in the 2030s declined, achieving a maximum area with approximately 82 × 10^4^ km^2^ under SSP5-8.5-2030s, and increased in all scenarios in the 2050s, achieving a maximum of approximately 104 × 10^4^ km^2^ under SSP1-2.6-2050s or SSP2-4.5-2050s. The overlap distribution of *L. temulentum* and *A. tauschii* in the 2030s and 2050s was similar to the current distribution, mainly distributed in East, Central, and eastern Southwest China. The remaining area of *L. temulentum* increased in the 2030s and 2050s in all scenarios except SSP5-8.5-2050s, achieving a maximum under SSP2-4.5-2030s (56 × 10^4^ km^2^) and SSP2-4.5-2050s (41 × 10^4^ km^2^), whereas *A. tauschii* decreased in the 2030s and 2050s in all scenarios except SSP 5-8.5-2050s, achieving a maximum under SSP1-2.6-2030s (92 × 10^4^ km^2^) and SSP5-8.5-2050s (149 × 10^4^ km^2^). The overlap change of *L. temulentum* and *A. tauschii* decreased by 21 × 10^4^, 14 × 10^4^,8 × 10^4^ km^2^ in the 2030s under SSP1-2.6, SSP2-4.5, and SSP5-8.5, respectively, and increased in the 2050s by 14 × 10^4^, 14 × 10^4^,1 × 10^4^ km^2^ for the same scenarios.

### 3.6 Environmental variables

We screened most significant environmental variables of *L. temulentum* and *A. tauschii*, through percent contribution and “Jackknife method”. For *L. temulentum*, human influence index (HII, 43.6%), mean temperature of coldest quarter (bio11, 27.7%), and precipitation of coldest quarter (bio19, 19.8%) were screened (**Figure S2** and **S5**). For *A. tauschii*, human influence index (HII, 33.8%), temperature seasonality (standard deviation×100) (bio4, 30.8%) and annual mean temperature (bio1, 14.7%) were screened (**Figures S2** and **S6**).

The probability of presence of significant environmental factors in *L. temulentum* and *A. tauschii* is shown in the response curve (**Figure S7**). This value is greater than 0.5, which indicates that the range of environmental variables is more suitable for the development of *L. temulentum* and *A. tauschii.* For *L. temulentum*, mean temperature for the coldest quarter was −8.52–16.53 °C, precipitation of coldest quarter was 72.64–914.52 mm, and the influence of human influence index on *L. temulentum* growth was increasing. For *A. tauschii*, the influence of human influence index was increasing, temperature seasonality was 7.68–10.57 °C, and annual mean temperature was 8.88–26.44 °C.

## 4 Discussion

Owing to the high reproductive rates, the toxicity of *L. temulentum*, and the pesticide resistance of *A. tauschii*, both species are regarded as significant IAPs in China. They threaten to decrease crop yield and wheat quality by robbing the soil of nutrients and competing with crops and hazard human health (Zhou et al., 2007; Thomas et al., 2011; Fang et al., 2015; Gao et al., 2021b). In this study, we identified the potential geographical distribution and niche overlap of *L. temulentum* and *A. tauschii* under the current climate and future scenarios in the 2030s and 2050s using the optimal MaxEnt model, which provides a basis for early warning and combined management.

### 4.1 Significant environmental variables

The most critical environmental variable for the distribution patterns of *L. temulentum* and *A. tauschii* was HII. Previous studies have confirmed that the established and spread of IAP populations in a new environment are closely linked to human activities, such as trade, tourism, transport, and human population density (McKinney, 2001; Pyšek et al., 2010). Another study indicated that the niche width of non-native plants increases with human activities (Irl et al., 2021). Non-native plants are carried by humans through travel and trade transportation, leaving their native region to novel environment (Boivin et al., 2016; Kueffer, 2017). Once, these invasive plants establish themselves and the populations spread, they integrate into ecosystems and ultimately have a negative influence on native species in the region (Kueffer, 2017). In our results, the value of HII increased concomitantly with increases in the probability of the species presence, revealing that HII played an important role and possibly influenced the geographical distribution of *L. temulentum* and *A. tauschii.*

In addition, for *L. temulentum*, bio11 and bio19 were the greatest contributions among 14 environmental variables. Low temperatures and high soil moisture are the conditions needed for the germination and growth of *L. temulentum* (Sagar et al., 1979). Vernalization can accelerate flowering, although *L. temulentum* seeds are dormant at 2 °C, but this did not reduce the seeds’ viability (Steiner and Ruckenbauer, 1995). When the temperature is higher than 5 °C, *Lolium* spp. develop a ‘stored growth’ potential that accelerate the extension of leaves (Peacock, 1975; Fournier et al., 2005). Our results showed that bio11 was −8.52–16.53 °C, which was consistent with the characteristic of low-temperature growth. Temperature, precipitation, and HII were vital environmental variables for *L. temulentum* growth. One study indicated that a mean annual precipitation of 400 to 1200 mm was an optimal development condition (CABI, 2022). Furthermore, we showed that approximately 900 mm of bio19, was optimum to establish populations for *L. temulentum.* For *A. tauschii*, and the greatest contributions among 17 environmental variables were bio4 and bio1, which were selected to predict the geographical distribution of *A. tauschii* in the optimal MaxEnt model. Previous studies have shown that the germination temperatures of *A. tauschii* range from 5 to 35 °C (Fang et al., 2015). Our results showed that the optimal range of temperature seasonality and annual mean temperature was consistent with this characteristic. The climatic characteristics of the living species were the dominant factors for population regeneration and establishment (Hansen et al., 2018).

### 4.2 Invasive historical reconstruction

Non-native plants that establish populations in novel environments undergo a two-stage process (Aikio et al., 2010). The first phase is the lag phase, when the occurrence of the invasive plants increase slowly or do not change at all, and the second is increase phase, species occurrence increase markedly (Crooks and Soulé, 1999). The lag phase is speculated to be closely linked to an adaption period invasive genotypes, abrupt invasion mechanisms, and environmental switches (Aikio et al., 2010). The invasive historical reconstruction of *A. tauschii*, showed that species occurrence increased slowly over 30 to 40 years after it invaded China, followed by population outbreak in 2000. This is exemplary of the two-stage process shown by the initial lag phase followed by a state of rapid growth. Alternatively, for *L. temulentum*, there was no obvious pattern, just a moderate increase. It has been suggested that this may have been influenced by sampling bias, such as incomplete survey techniques and monitoring (Delisle et al., 2003). All occurrences of *L. temulentum* and *A. tauschii* increase as awareness of IAPs increase and this is attributed to the progressive increases in the search and documentation of species occurrence (Cousens and Mortimer, 1995). *Lolium temulentum* and *A. tauschii* are critical IAPs in China, and their occurrence could continue to increase in the future.

### 4.3 Geographical distribution and ecological niche

The potential geographical distribution of some invasive plants will shrink, whereas others will expand with climate change (Tu et al., 2021; Zhang et al., 2021b). The suitable areas of *Ageratina adenophora* in China would decrease under the 2050s, whereas increased in the 2090s (Zhang et al., 2022). In this study, the total trend in suitable areas for *L. temulentum* increased from the current climate to future scenarios (SSP1-2.6, SSP2-4.5, and SSP5-8.5) in the 2030s and 2050s. This result is similar to other studies on the potential geographical distribution of non-invasive plants; for example, suitable areas of *Cenchrus ciliaris* increased under SSP1-2.6 and SSP2-4.5-2050s, and for *Spartina anglica* the suitable areas increase under SSP5-8.5-2100 in Northern Europe (Borges et al., 2021; Siller-Clavel et al., 2022). IPCC has indicated that global warming is likely to increase by 1.5 °C from 2030 to 2052 at the current rate (IPCC, 2021). The radiative forcing gradually increased in SSP1-2.6, SSP2-4.5, and SSP5-8.5, reaching 2.6, 4.5, and 8.5 W/m^2^, respectively, indicating that global CO_2_ concentrations are rising. *Lolium temulentum* is a C_3_ plant that strengthens photosynthesis to advance nutrient absorption, facilitating the development of a rise in CO_2_ concentrations, so the predicted suitable areas of *L. temulentum* will likely increase in future scenarios (Lush, 1976). Previous studies have shown that the suitable areas for some invasive plants shrank but still had a significant invasion risk, such as *Xanthium italicum* (Zhang et al., 2021b). This result is consistent similar with the predicted suitable areas for *A. tauschii* in the 2030s in this study, but it ultimately ended up increasing in the 2050s. Furthermore, the IPCC report indicated that the yield of some crops that grow at different latitudes, such as wheat, maize, and sugar beet, have been influenced by climate change in recent years (IPCC, 2021). We hypothesize that the change in the potential geographical distribution of *L. temulentum* and *A. tauschii* is related to this.

Plants can change their physiological characteristics to migrate to a new habitat or respond more suitably to climate change (Davis and Shaw, 2001).However, not all species have the ability to evolve adaptations that respond to rapid climate change; therefore, migration is considered a critical pathway to address this issue (Etterson and Shaw, 2001; Corlett and Westcott, 2013). some non-native plants, such as *Alternanthera philoxeroides* and *Ambrosia artemisiifolia*, are extremely adaptable and can migrate whereas others cannot with climate change (Tu et al., 2021). Previous studies have shown that climate change plays a critical role in range-shifting invasive plants, which may be geographically stable with climate warming, or shifting to different directions in future scenarios (Allen and Bradley, 2016; Rockwell-Postel et al., 2020). Our data show that the centroid of *A. tauschii* is primarily located in Henan from the current climate and in future scenarios, and it is unable to keep pace with climate change. However, *L. temulentum* transfers to the north and to different provinces under future scenarios, which indicates high adaptability to climate change.

Niche overlap is deemed to be ecological species that exhibit biological characteristics and ecological adaptability, including similarities in resource utilization and species competition (Jing et al., 2015). When the value of niche width is greater, it indicates greater adaptability to the environment and niche overlap (Wang et al., 2019). Our results demonstrate that *L. temulentum* and *A. tauschii* have a lot of similarities in resource utilization and geographical distribution, which may be due to the synergistic reduction in wheat quality. By calculating B1 and B2, it is evident that *L. temulentum* is more adaptable to the habitat of *A. tauschii.* The toxicity of *L. temulentum* threatens human and animal health, and the high reproduction rate and pesticide resistance of *A. tauschii* pose a significant threat of its spread. Therefore, some control measures have been considered and are proposed herein.

### 4.4 Control measures

We demonstrated that the current overlap of *L. temulentum* and *A. tauschii* is primarily distributed in Yunnan, Sichuan, Guizhou, Chongqing, Hunan, Jiangxi, Zhejiang, Shanghai, Hubei, Anhui, Jiangsu, Shandong, Henan, Shaanxi, Gansu, Shanxi, Hebei, Beijing, Liaoning, and Xinjiang, and this is consistent with previous study (Fang et al., 2013; CABI, 2022). Yunnan, Zhejiang, and province regions were predicted to be novel environments that exist at high invasive risk for *L. temulentum* and *A. tauschii.* Spreading occurs primarily from seed dispersal during wheat trade transport to other provinces in China, therefore it is quintessential to secure wheat breeding bases without *L. temulentum* and *A. tauschii* seeds to increase wheat quality and purity, and these acceptance are considered effective control measures (Terreix, 1968; Fang et al., 2013; Gong, 2022).

Furthermore, other studies have suggested that cultural control and chemical control are effective means for controlling *L. temulentum* and *A. tauschii*. Cultural control includes establishing long rotations periods that disturb the soil environment or using wheat seed cleaning screens (Ferrari et al., 1984). Another study demonstrated that sowing wheat 30 and 60 days later and hand weeding twice was effective in controlling *L. temulentum* (Angiras and Rana, 1990). Strengthening seed quarantine, crop rotation that changes the environment of weed populations, and artificially pulling weeds are effective ways to control *A. tauschii* (Yu et al., 2018). However, pulling weeds can be difficult because of the similarities between *L. temulentum* and wheat. Therefore, individuals should take measures to control *L. temulentum* in combination with pesticides. The application of triallate and metoxuron to the soil can effectively control *L. temulentum* development before sowing wheat (Stevens and Meyes, 1976). In addition, the pre-emergence of wheat applications of metribuzin or pendimethalin show substantial governance (Cairns et al., 1979). As for *A. tauschii*, mesosulfuron-methyl is considered an effective herbicide, whereas excessive spraying could restrain wheat development such that seedlings become yellow or weak (Wu et al., 2022). Therefore, the dose, proportion of inert ingredients, and spraying time of mesosulfuron-methyl are the important issues to consider to avoid herbicide pollution. Recent studies have shown that mesosulfuron-methyl is not a permanent control of *A. tauschii* growth, which have developed resistance in parts of China, including the northwest plain, central mountains, and northern coastal areas in Shandong (Gao, 2021).

## 5 Conclusions

*Lolium temulentum* and *A. tauschii* are the primary contributors to wheat loss in China. Climate warming and human activities have accelerated the invasion of these IAPs and the establishment of *L. temulentum* and *A. tauschii* in new habitats throughout China. Reconstructing the historical invasive progress of *L. temulentum* and *A. tauschii* in China showed that *L. temulentum* and *A. tauschii* spread to new environments that were suitable to their development and potential expansion. Based on the occurrence of the screened species and related environmental variables, the suitable habitat and overlap area of *L. temulentum* and *A. tauschii* were predicted using the optimal MaxEnt. Our results showed that the total trend in suitable areas of *L. temulentum* increased under SSP1-2.6, SSP2-4.5, and SSP5-8.5 in the 2030s and 2050s, whereas that of *A. tauschii* decreased. Meanwhile, the overlap areas of *L. temulentum* and *A. tauschii* decreased in the 2030s but increased in the 2050s, mainly in central and eastern China. The future centroid of *L. temulentum* transferred to higher latitudes, while *A. tauschii* transferred to lower latitudes compared to the current climate, which revealed that climate change played a critical role in geographical distribution. The results obtained from optimal MaxEnt provide new insights for long-term detection and management.

## Data availability statement

The original contributions presented in the study are included in the article/**Supplementary Material**. Further inquiries can be directed to the corresponding author.

## Author contributions

MY and HZ and: conception and design of the research. MY: acquisition of data, analysis and interpretation of data. MY and HZ: statistical analysis. HZ: drafting the manuscript. XX, RW, NY, LC, and W-XL: manuscript revision. All authors contributed to the article and approved the submitted version.
