# Supplementary material for: Assessing risk from invasive alien plants in China: Reconstructing invasion history and estimating distribution patterns of Lolium temulentum and Aegilops tauschii
Source: Front Plant Sci. 2023 Feb 2;14:1113567. doi: 10.3389/fpls.2023.1113567 (PMC9933513; doi:10.3389/fpls.2023.1113567)
Supplement: Supplementary file 1 [file DataSheet_1.docx]

Supplementary Material

# Supplementary Figures and Tables

## Supplementary Figures


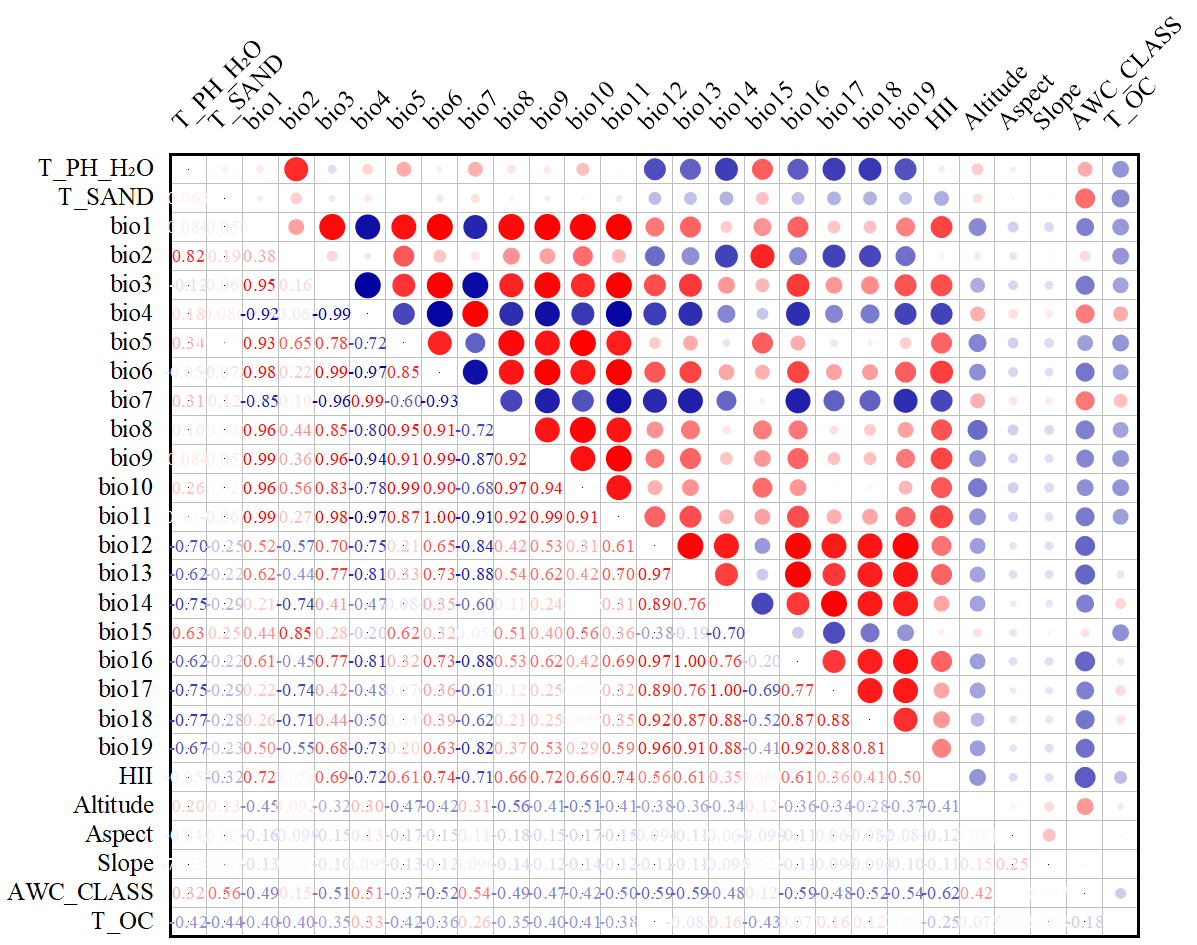


**Supplementary Figure 1.** Relation between 27 environmental variables


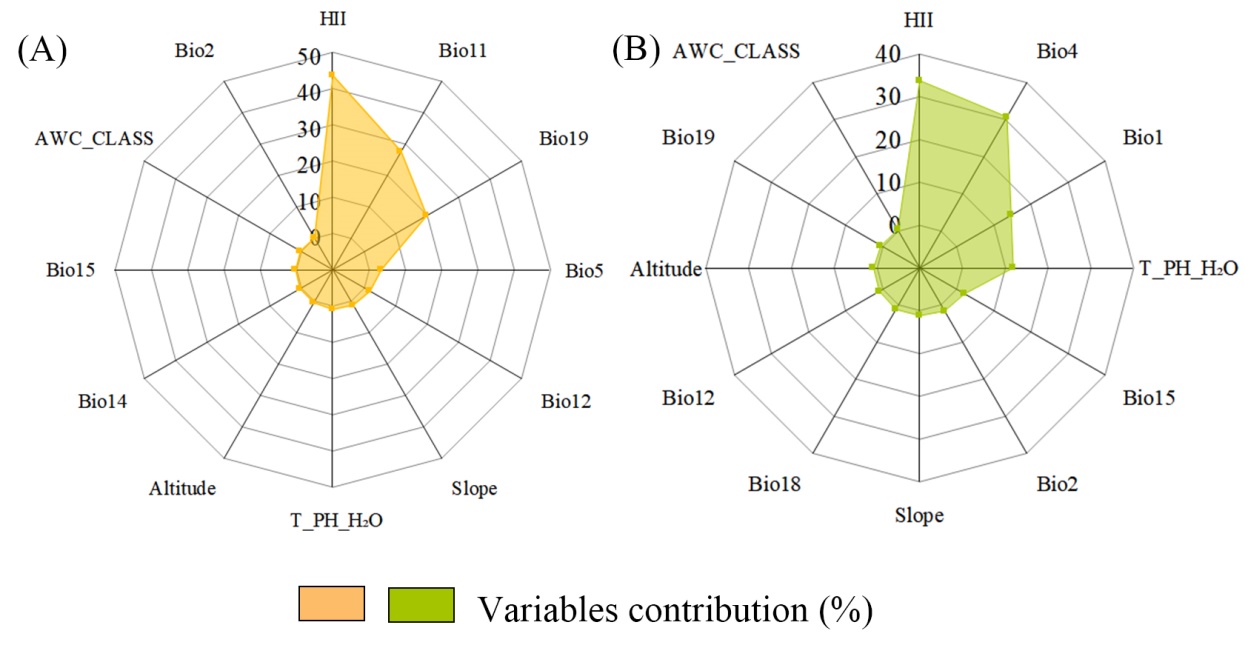


**Supplementary Figure 2.** The environmental variables contribution of (A) *Lolium temulentum* and (B) *Aegilops tauschii*.


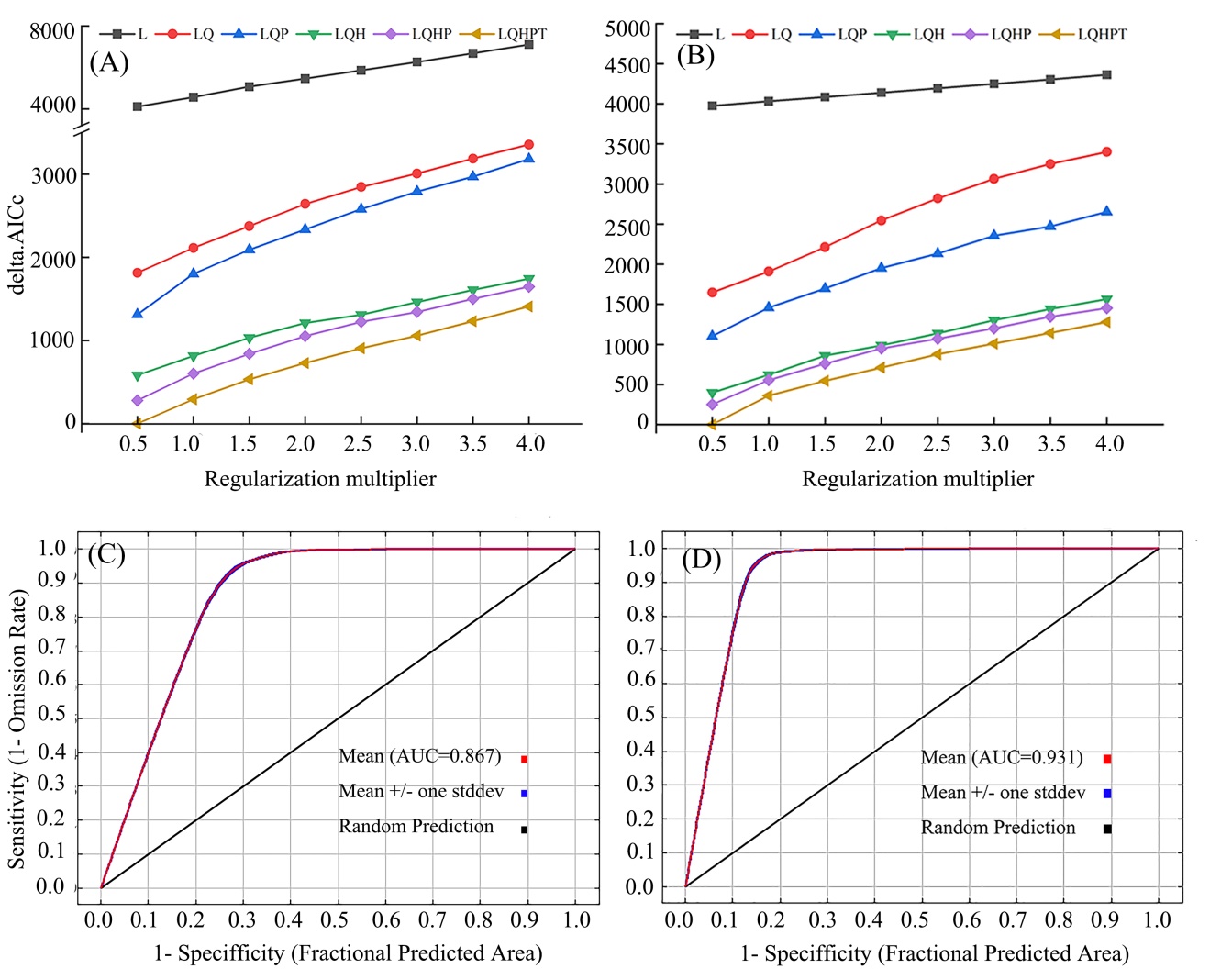


**Supplementary Figure 3.** The MaxEnt model calibration and AUC of *Lolium temulentum* and *Aegilops tauschii*. (A) and (C) were the optimal model parameter chosen for *L. temulentum* and the predicted model performance*.* (B) and (D) were the optimal model parameter chosen for *A. tauschii* and the predicted model performance*.* The optimal RM was set as 0.5, and FC was set as LQHPT for both when Dela.AICc=0.


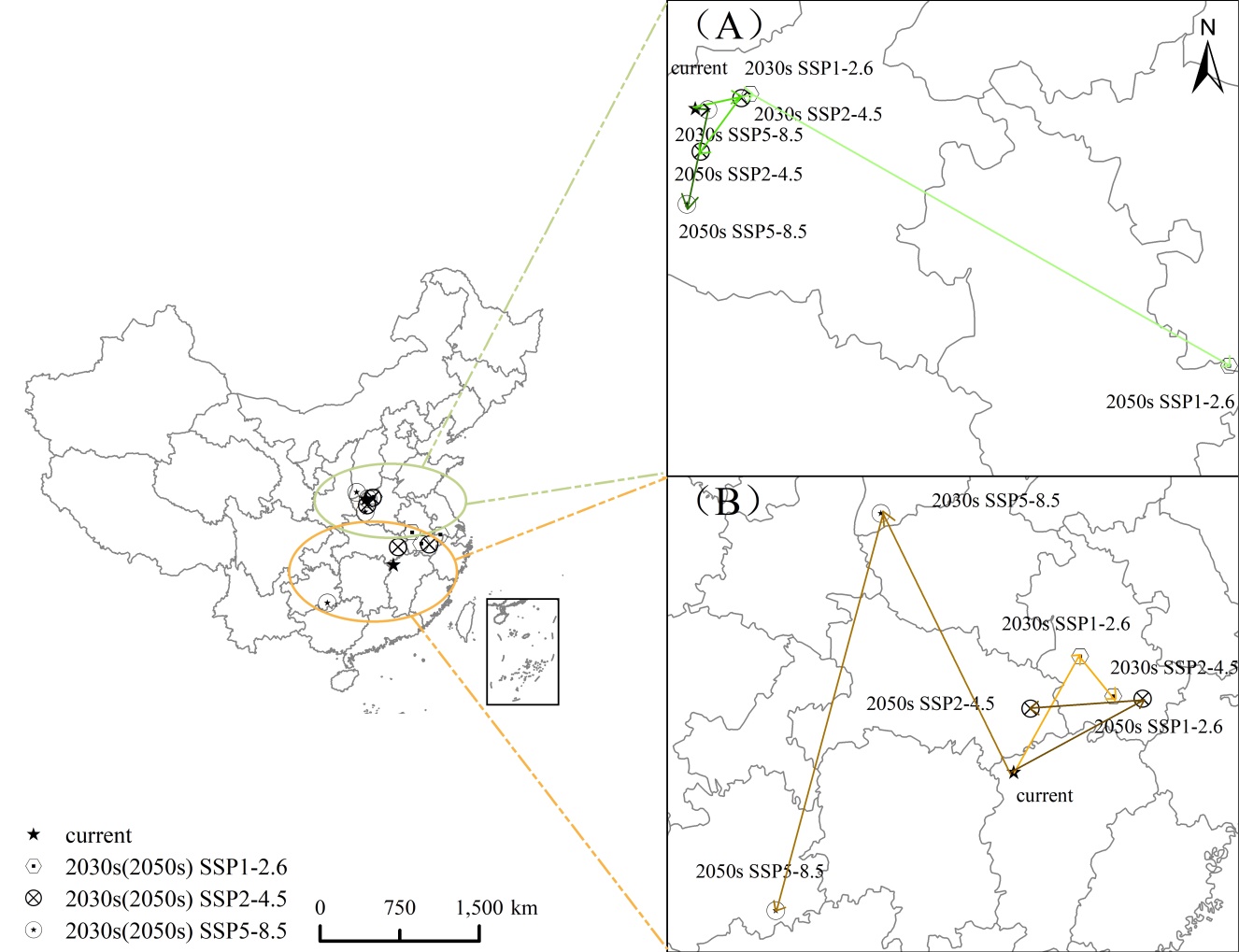


**Supplementary Figure 4.** The centroid distribution of (A) *Aegilops tauschii* and (B) *Lolium temulentum* under the current climate and future scenarios in the 2030s and 2050s, including SSP1-2.6, SSP2-4.5, and SSP5-8.5.


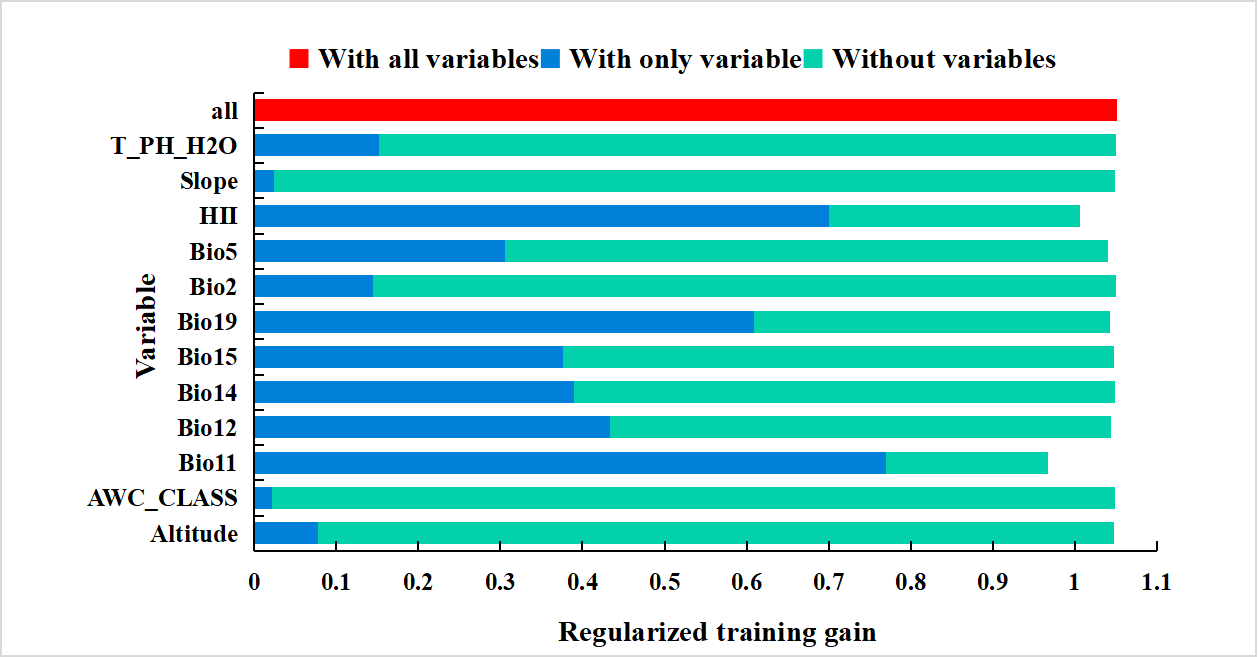


**Supplementary Figure 5.** Importance of environmental variables of *Lolium temulentum.*by “Jackknife method”


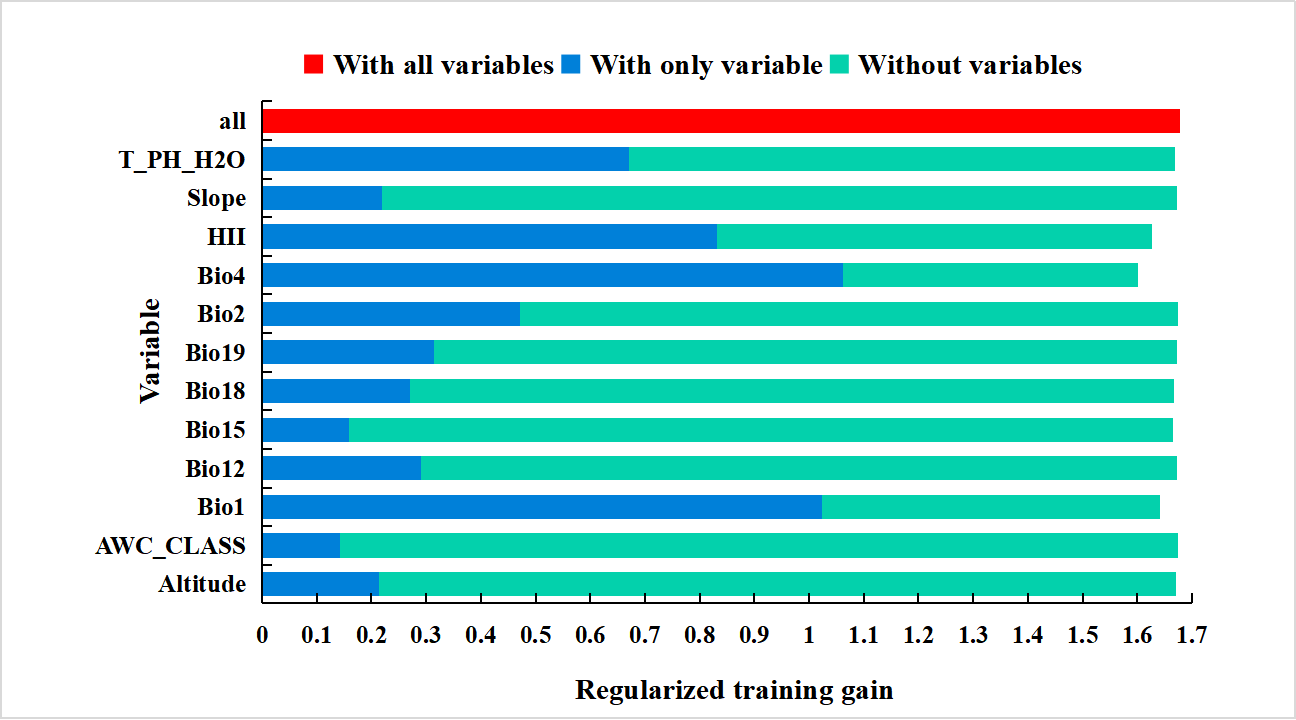


**Supplementary Figure 6.** Importance of environmental variables of *Aegilops tauschii.*by “Jackknife method”


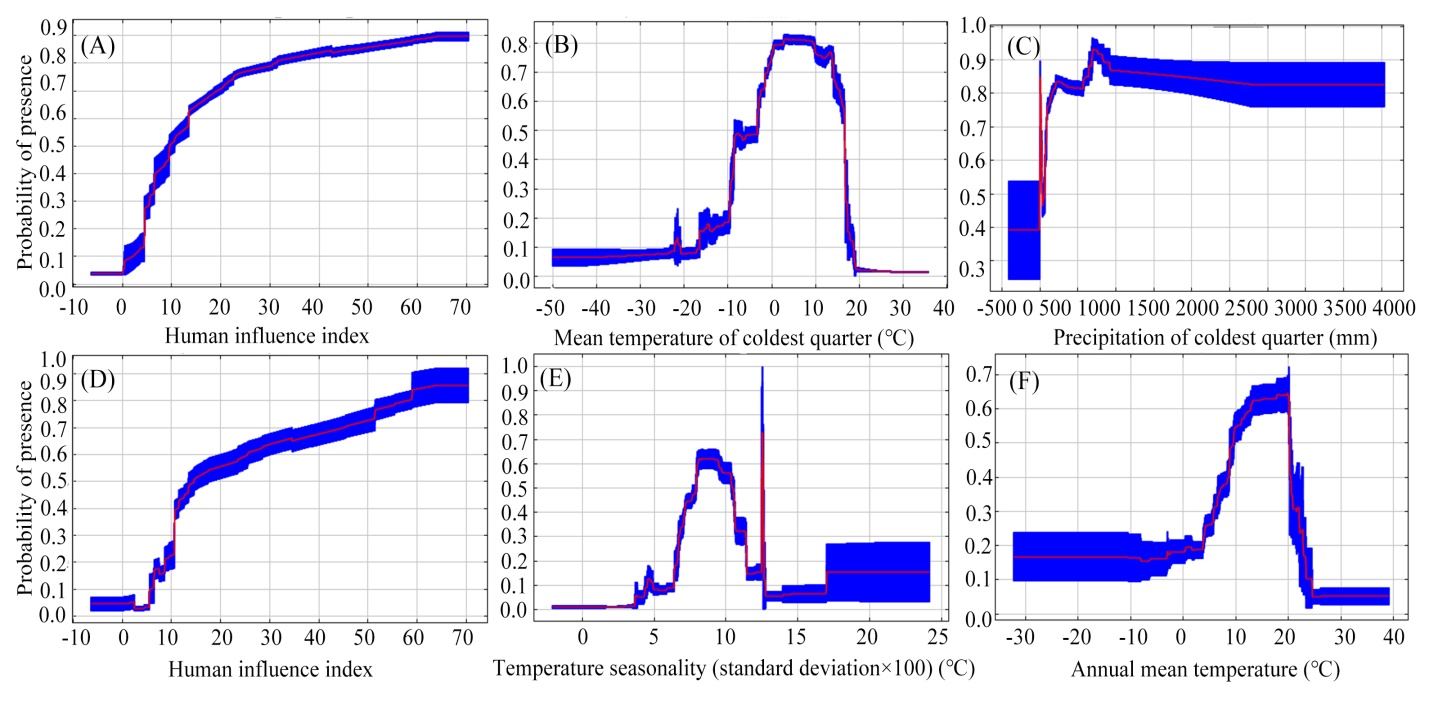


**Supplementary Figure 7.** Response curve of the most key environment variables of (A-C) *Lolium temulentum* and (D-F) *Aegilops tauschii*.

**Supplementary Tables 1.** Environmental variables for the potential geographical distribution of *Lolium temulentum.*

| Variable | Description | Unit |
| --- | --- | --- |
| bio1 | Annual mean temperature | ℃ |
| bio2 | Mean diurnal temperature range | ℃ |
| bio3 | Isothermality (Bio2/Bio7)×100 | - |
| bio4 | Temperature seasonality (standard deviation×100) | ℃ |
| bio5 | Max temperature of warmest month | ℃ |
| bio6 | Min temperature of coldest month | ℃ |
| bio7 | Temperature annual range | ℃ |
| bio8 | Mean temperature of wettest quarter | ℃ |
| bio9 | Mean temperature of driest quarter | ℃ |
| bio10 | Mean temperature of warmest quarter | ℃ |
| bio11 | Mean temperature of coldest quarter | ℃ |
| bio12 | Annual precipitation | mm |
| bio13 | Precipitation of wettest month | mm |
| bio14 | precipitation of driest month | mm |
| bio15 | Precipitation seasonality (coeffcient of variation×1 | - |
| bio16 | Precipitation of wettest quarter | mm |
| bio17 | Precipitation of driest quarter | mm |
| bio18 | Precipitation of warmest quarter | mm |
| bio19 | Precipitation of coldest quarter | mm |
| Altitude | Altitude | m |
| Slope | Slope |  |
| Aspect | Aspect | % |
| AWC_CLASS | Soil Available Water Content Range |  |
| T_OC | Topsoil Organic Carbon | % weight |
| T_PH_H_2_O | Topsoil pH (H_2_O) | -log(H+ ) |
| T_SAND | Topsoil Sand Fraction | % wt |
| HII | Human Influence Index |  |
